# Drastic underestimation of amphipod biodiversity in the endangered Irano-Anatolian and Caucasus biodiversity hotspots

**DOI:** 10.1038/srep22507

**Published:** 2016-03-01

**Authors:** Ahmad-Reza Katouzian, Alireza Sari, Jan N. Macher, Martina Weiss, Alireza Saboori, Florian Leese, Alexander M. Weigand

**Affiliations:** 1 School of Biology and Centre of Excellence in Phylogeny of Living Organisms, University of Tehran, Tehran, Iran; 2 Aquatic Ecosystem Research, University of Duisburg-Essen, Essen, Germany; 3 Department of Plant Protection, Faculty of Agriculture, University of Tehran, Karaj, Iran; 4 Centre for Water and Environmental Research, University of Duisburg-Essen, Germany; 5 Department of Animal Ecology, Evolution and Biodiversity, Ruhr University Bochum, Bochum, Germany

## Abstract

Biodiversity hotspots are centers of biological diversity and particularly threatened by anthropogenic activities. Their true magnitude of species diversity and endemism, however, is still largely unknown as species diversity is traditionally assessed using morphological descriptions only, thereby ignoring cryptic species. This directly limits evidence-based monitoring and management strategies. Here we used molecular species delimitation methods to quantify cryptic diversity of the montane amphipods in the Irano-Anatolian and Caucasus biodiversity hotspots. Amphipods are ecosystem engineers in rivers and lakes. Species diversity was assessed by analysing two genetic markers (mitochondrial *COI* and nuclear 28S rDNA), compared with morphological assignments. Our results unambiguously demonstrate that species diversity and endemism is dramatically underestimated, with 42 genetically identified freshwater species in only five reported morphospecies. Over 90% of the newly recovered species cluster inside *Gammarus komareki* and *G. lacustris*; 69% of the recovered species comprise narrow range endemics. Amphipod biodiversity is drastically underestimated for the studied regions. Thus, the risk of biodiversity loss is significantly greater than currently inferred as most endangered species remain unrecognized and/or are only found locally. Integrative application of genetic assessments in monitoring programs will help to understand the true magnitude of biodiversity and accurately evaluate its threat status.

Biodiversity is a key resource of our planet providing important ecosystem functions thereby ensuring sustainable life on Earth^1^,^2^,^3^. However, the dramatic loss of biodiversity is proceeding at a striking pace and has become a major concern for human well-being^4^,^5^,^6^. Environmental legislation and management actions have been implemented at both international and national levels to counteract biodiversity loss. The establishment of national parks, the convention on biological diversity, the United Nations decade on biodiversity from 2011–2020 or the declaration of so-called biodiversity hotspots are just a few instances of such recognition. The concept of biodiversity hotspots involves defined regions of high conservation priority referring to both, a high threat to their natural intact vegetation (NIV) and an exceptional richness of endemic (by definition: vascular plant) species^7^,^8^,^9^. On a global scale, 35 biogeographic regions qualify as biodiversity hotspots^10^, covering 17.3% of the Earth’s land surface (excluding Antarctica) and being home for more than 70% of the known animal fauna^10^,^11^. Biodiversity hotspots are critically prone to biodiversity loss as they harbour many endemic species, but likewise unique ecosystems and gene variants^7^,^8^. Despite of their negligible surface area (<1% on a global scale), freshwater ecosystems are of particular concern as they provide habitat to an over-proportionally great number of species^12^. The efficiency of traditional morphology-based methods in monitoring biodiversity changes is widely accepted, however, it is also well known that these methods lack the ability to identify cryptic species and cannot inform on the loss of genetic variation. Hence, they underestimate the “true” biological diversity present in an ecosystem, which adds an additional level of uncertainty for monitoring and management actions^13^. Therefore, it is of utmost necessity to employ fast and efficient tools to monitor the ongoing biological diversity loss in particular in freshwater ecosystems, and genetic tools have been proven to be extremely effective^14^,^15^,^16^,^17^,^18^,^19^,^20^,^21^,^22^. One ecologically highly important animal group for which recent studies have shown that species assignments are difficult is amphipod crustaceans^23^,^24^,^25^,^26^,^27^. They play an important functional role in freshwater food webs^28^,^29^ by i) serving as prey for fishes^30^,^31^, ii) acting as intermediate and definitive hosts for parasites^29^,^32^,^33^, and iii) regulating organic litter breakdown^34^, and must be considered as ecological keystone engineers.

Here, we used freshwater amphipods of the genus *Gammarus* sampled from freshwater ecosystems of two mountain ranges in the Caucasus (Northern slopes of the Alborz Mountains, Iran) and Irano-Anatolian biodiversity hotspot (Zagros Mountains and southern slopes of the Alborz, both Iran) as a model to test whether the true species diversity is accurately reflected by the present morphology-based estimates. To this aim, amphipod species were genetically surveyed and linked to morphologically known species for the broader region of Iran, with three and 16 morphologically defined species present in the Alborz and Zagros Mountains, respectively^35^. We expect our molecular-based approach to reveal a more realistic pattern of higher biological diversity in those two critically endangered biodiversity hotspots. Furthermore, based on recent data from other regions^23^,^24^,^25^,^26^,^27^,^36^ we expect that several of the reported widespread species will consist of regional endemics.

## Results

### Species diversity based on morphology

The following freshwater species have been identified morphologically: *Gammarus lacustris* s. str. Sars^37^, (4 specimens from 2 localities), *Gammarus* cf. *lacustris* Sars^37^, (=*G. lacustris* complex) (12 specimens, 6 localities), *Gammarus lordeganensis* Khalaji-Pirbalouty and Sari^38^, (7 specimens, 3 localities), *Gammarus balcanicus* Schäferna^39^, (1 specimen, 1 locality), *Gammarus hegmatanensis* Hekmatara *et al.*^40^, (2 specimens, 1 locality) and *Gammarus* cf. *komareki* Schäferna^39^, (=*G. komareki* complex) (154 specimens, 49 localities) (Table 1; for type localities see Supplementary Table 1). The latter was by far the most abundant morphospecies in the studied freshwater systems. The designation “cf.” was applied to species when taxonomic assignment was uncertain as many of the surveyed individuals showed ambiguous diagnostic characters, i.e. largely corresponding to the respective morphospecies description but not allowing explicit species assignment. Furthermore, two brackish water species were collected serving as outgroups for the molecular analyses: *Obesogammarus acuminatus* Stock *et al.*^41^, (2 specimens, 1 locality) and *Gammarus aequicauda* (Martynov)^42^ (2 specimens, 1 locality).

### Species diversity based on molecular data

**Figure 1:**
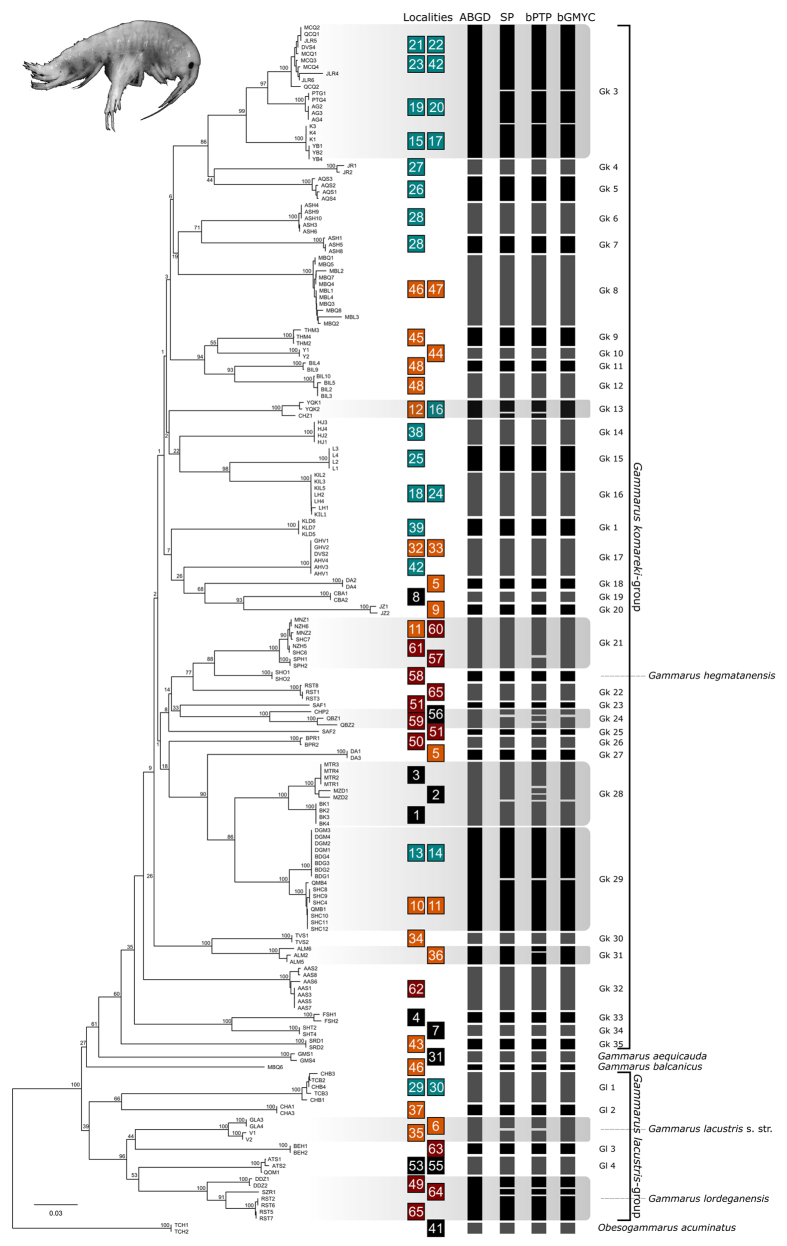
Neighbor-Joining tree visualizing the results of the four different molecular species delimitation methods based on *COI*. ABGD: Automatic Barcode Gap Discovery; SP: reversed Statistical Parsimony; bPTP: Bayesian Poisson tree process model; bGMYC: Bayesian General Mixed Yule-Coalescent model. Localities for each species are color-coded in green (Alborz Mountains, in Caucasus biodiversity hotspot), orange (Alborz, Irano-Anatolian), red (Zagros, Irano-Anatolian) and black (Central Iranian Plateau or brackish water localities at the Caspian Sea; 31 and 41). Bootstrap support values are provided at the branches. Gk1-35: Cryptic species within the *G. komareki* complex; Gl1-4: Cryptic species within the *G. lacustris* complex. The depicted specimen is a representative of the *Gammarus komareki* complex.

The final *COI* alignment included 184 specimens trimmed to 465 bps. All *COI*-sequences were checked against the NCBI database for possible contaminations, which were not present. The four conceptually different molecular species delimitation methods revealed 44–58 groups. The Automatic Barcode Gap Discovery (ABGD) approach delineated 44 groups (Fig. 1) with a proposed barcoding gap at 7.9% Kimura-2-Parameter (K2P) genetic distance. Of the 44 groups, 35 were morphologically identified as belonging to the *Gammarus komareki* complex and six to the *G. lacustris* complex. The reversed Statistical Parsimony (SP) approach revealed 53 groups. Among those groups, 41 were within the *G. komareki* complex and nine belonged to *G. lacustris*. The Bayesian General Mixed Yule-Coalescent (bGMYC) model split the dataset into 51 groups, with 40 and eight within the *G. komareki* and *G. lacustris* species complex, respectively. Finally, the delimitation approach based on the Bayesian implementation of the Poisson Tree Process (bPTP) model yielded the largest number of groups (58). Of those, 46 were within the *G. komareki* complex and nine in *G. lacustris*. *Obesogammarus acuminatus*, *G. balcanicus, G. aequicauda* and *G. hegmatanensis* were identified as single delineated groups each in all four approaches. *Gammarus lordeganensis* and *G. hegmatanensis* were always found to be part of the *G. lacustris* and *G. komareki* complex, respectively.

As the four delimitation approaches yielded different numbers of groups, we here refer to the 44 groups revealed by AGBD as the most conservative approach. This is reasonable since i) by referring to the lowest species estimates, we follow a most parsimonious decision avoiding an over-splitting of lineages and an associated overestimation of amphipod species richness and endemism, ii) none of the lineages proposed by ABGD is lumped by any of the other three methods, and iii) 35 of the ABGD lineages (80%) are congruently detected in all four approaches. Four more lineages are supported by ABGD and at least one other delimitation approach (9%). Only five lineages (11%) are exclusively found by ABGD but distinctly split in the other three approaches.

Six of the 44 putative species could be morphologically linked to a species name: *Gammarus hegmatanensis*, *G. balcanicus*, *G. lacustris* s. str., *G. lordeganensis*, *G. aequicauda* and *Obesogammarus acuminatus*, comprising four freshwater and two brackish water species, respectively. The remaining 38 groups represented cryptic species within the two species complexes of *G. komareki* (n = 34) and *G. lacustris* s. l. (n = 4). Groups within the *G. komareki* complex were consistently named Gk1 to Gk35, with Gk1 and Gk2 already introduced by Hou *et al.*^43^. Only Gk1 was found in our study. Groups of the *G. lacustris* complex were named Gl1-Gl4.

In order to test for congruency with the *COI* delimitation results, nuclear 28S rDNA data was successfully obtained for 87 specimens covering all putative species except Gl4. The 28S alignment was trimmed at both ends to a final length of 798 bp for all but four sequences, which were shorter at the 3′ end (YB1 = 795 bp, BIL5 = 789 bp, QCQ1 = 765 bp and JLR5 = 759 bp). ABGD groupings are supported by nuclear data in most of the cases (36 out of 43; 84%), except: i) the specimens L1, LH2, SRD1, SRD2 and KLD6 showed a highly similar 28S sequence but belonged to four different ABGD (*COI*) groups, ii) the specimen NZH5 had a highly distinct 28S sequence (>10 mutations difference) when compared with the nuclear sequences of the six other specimens of the ABGD group Gk21 for which nuclear data was obtained, iii) the two specimens of Gk4 (JR1 and JR2) possess nuclear sequences with seven mutations difference, and iv) the three specimens of the sampling site Biledargh (BIL5, BIL9 and BIL10) clustered into two *COI*-groups (Gk11, Gk12), but all exhibit 28S sequences with 3–4 mutations difference, not allowing a clear distinction of the two *COI*-groups with nuclear data (Supplementary Fig. 1). However, the latter two cases do not contradict the initial delimitation results.

## Discussion

It is a generally accepted fact that biodiversity is in decline due to anthropogenic land use and climate change. However, the true magnitude of biodiversity loss is a topic of central debate. Our results underscore the benefit of molecular tools in capturing the “true” biological diversity present in an ecosystem. The montane freshwater amphipod fauna of the Alborz and Zagros Mountains, situated within the biodiversity hotspots of the Caucasus and Irano-Anatolian region, comprises over an order of magnitude more species than previously known, meaning that most species were overlooked by traditional morphology-based assessments. In line with our expectations, the 42 gammarid freshwater species detected in this study can be linked to just five morphospecies. More than 90% of the found species are cryptic and cluster within the two species complexes of *Gammarus komareki* (34 cryptic species) and *Gammarus lacustris* (four cryptic species). Only four freshwater species (10%) could be unambiguously linked with morphology, i.e. a single morphospecies comprising a single genetically identified species: *Gammarus hegmatanensis*, *Gammarus balcanicus*, *Gammarus lacustris* s. str. and *Gammarus lordeganensis.* Although our sampling covers 154 freshwater sites, there exists an obvious geographical sampling bias towards freshwater systems in the Alborz and Northern Zagros Mountains. In general, sampling effort affects the number of species and the level of intraspecific genetic diversity to be discovered^44^,^45^,^46^ and it is likely that further unrecognized members of both widespread species complexes, *G. komareki* and *G. lacustris*, also occur in the Southern Zagros Mountains. *Gammarus balcanicus* might be another candidate species complex comprising cryptic lineages within the studied region, as it has been revealed for montane freshwater systems in Europe^25^. However, our sampling was not appropriate to assess hidden species diversity for this species in Iran.

The observed high level of cryptic species diversity becomes even more intriguing when compared to the number of amphipod freshwater species previously reported for the study area, specifically for the Alborz Mountains at the junction of the Caucasian and Irano-Anatolian biodiversity hotspots. So far, three species have been reported from that region, however our results imply that species diversity is drastically higher, i.e. by more than one order of magnitude. After integration of the amphipod species data presented in this study with the known faunal record, the Alborz Mountains harbor 34 species and the Zagros Mountains 25 species (16 previously reported species), respectively. The observed pattern of a high number of unrecognized species is consistent with other studies on montane freshwater amphipod communities in Europe (e.g.^25^,^36^). On the one hand, many of the newly recognized species of the *G. komareki* and *G. lacustris* complexes fit to the assumed intraspecific morphological variability of their respective type species. On the other hand, some species demonstrate substantial morphological differences, e.g. in the degree of setation of the telson/second antenna, shape and size of the gland cone and shape of the third epimeral plate. As already noted, morphology alone often does not seem to be sufficient in describing *Gammarus* species (e.g.^24^,^47^,^48^,^49^). For each new species, taxonomists have to question whether the (sometimes ambiguous) combination of conserved and variable morphological traits are diagnostic or reflect the natural intraspecific variability of a species.

The delimitation of species based solely on molecular data likewise can sometimes also be problematic^50^. In general, single-gene approaches are error-prone if incomplete lineage sorting or introgression occur. Furthermore, single threshold delimitation estimates (e.g. genetic distance values, transition points or connection limits) can also be misleading when different species exhibit variable diversification rates. Therefore, to interpret results more carefully, a suitable analytical framework based on more than one gene and including a combination of delimitation approaches should be applied. In our study, the majority of species is supported by both mtDNA and nuclear data (84%) and by all four delimitation approaches (80%). There are many possible reasons that can lead to the discrepancies between different delimitation methods. However, in our case these discrepancies are mostly likely due to the known tendency of coalescent-based approaches (i.e. PTP and GMYC) to overestimate the number of lineages if only a few specimens per species are investigated and/or the proportion of singletons in the dataset is high^51^,^52^. Both these scenarios lower the portion of coalescing lineages in the analysis and produce younger transition point estimates, which will finally result in the delimitation of more recently diverged lineages. Incongruencies between both molecular markers can be likely attributed to the retention of ancestral polymorphisms (groups Gk1, 11, 12, 15, 16 and 35), which is fourfold more likely in nuclear as compared to mitochondrial genes, and to an mtDNA introgression event (specimen NZH5, group Gk21). For the future, an integrative taxonomic approach is needed to delimit and describe species with independent lines of evidence.

While the number of unrecognized species already documents the potential of molecular methods to rapidly quantify the *status quo* and threat status of a region, knowledge about a high level of unrecognized biodiversity is only the first step. The inferred patterns on species distributions add another layer of information. Contrary to the assumed wide distribution of some amphipod species present within the study region (e.g. *G. komareki* and *G. lacustris*), 69% of the genetically identified species only occurred at a single site. Even localities separated by only a few tens of kilometers were most often inhabited by distinct species. In cases where a species was present at multiple sites, localities were generally in close geographical proximity and/or a clear phylogeographical pattern with exclusive spatial lineages exists. Only specimens of a single group, i.e. Gk21, occurred in both the Alborz and Zagros mountain ranges. Passive transportation by humans, e.g. between the abundant trout farms, or by birds such as the mountain stream inhabiting dipper (*Cinclus cinclus* Linnaeus, 1758) may be responsible for the presence of this species in streams approximately 800 km apart. An increased sampling may reveal this species to be even more common and/or other more widespread species.

The occurrence of such a high level of narrow endemism as reported here can be explained by a combination of two evolutionary processes. First, diversification rate can be increased through the emergence of new ecological opportunities in general but specifically in gammaridean amphipods^43^. In particular, the transition from a saline to a freshwater environment is regarded as an evolutionary trigger for increased diversification rates observed in freshwater gammarids^43^. Second, when compared to marine habitats, freshwater ecosystems are much more isolated. This micro- and macro vicariance is likely the main driver further accelerating diversification of lineages. A similar scenario may be likely for marine *Gammarus*-ancestors from the Paratethys Sea colonizing freshwater habitats in Iran. The biogeographic setting of the studied mountain regions promote the formation of narrow endemism as rivers of the Alborz Mountains predominantly flow either north- or southwards and thus either directly end up in the Caspian Sea (northern slopes) or dry out in the Central Iranian Plateau and Dasht-e Kavir desert (southern slopes). The latter is also true for rivers in the eastern Zagros Mountains^53^. Hence, after the uplift of both mountain ranges during the Miocene and Pliocene, multiple hydrologically separated freshwater habitats were available for initial colonization, population differentiation and finally species diversification^43^. At this point, it has to be noted that our sampling scheme along both mountain ranges (with only a few specimens analyzed per sampling site) systematically favors the discovery of narrow endemism, in particular the degree of species found at a single site. A denser sampling (i.e. within a single river or catchment) may reveal broader distribution ranges of many species. The results, however, also suggest that further sampling, especially in yet sparsely sampled areas or during different seasons at temporally desiccated sites, will most likely reveal more endemic species.

Knowledge about the distinct pattern of small-scale endemism in freshwater amphipod species is of paramount importance for conservation and management strategies: since these amphipods primarily demonstrate a shredding feeding type, they will heavily rely on coarse organic material input, e.g. provided by the NIV^54^. As such, species are most likely co-affected by the continuing rapid decline of NIV observed for both biodiversity hotspots^55^. Considering the high probability that the rate of narrow endemism found for Iranian freshwater amphipods is the rule rather than the exception, this situation becomes even more severe. Endeavors in protecting and sustaining the NIV and accompanied faunal biological diversity within biodiversity hotspots should become another prime target.

Freshwater ecosystems and their biodiversity are endangered due to many factors, such as habitat degradation, overexploitation, flow modification and water pollution^56^. We showed that montane freshwater ecosystems in two highly endangered biodiversity hotspots harbor a drastically underestimated amphipod diversity, both at the species and genetic level. Many of the newly discovered species comprise narrow range endemics rather than widespread lineages. Although species extinction is globally perceived to a high degree, the dimension of biological diversity loss may be even greater than currently inferred given that most endangered species may still be unrecognized and/or only locally found. Finally, our study provides further support for the urgently needed and integrative implementation of genetic monitoring strategies^57^, which can make a plethora of information on the *status quo* and threat status of biodiversity, from the level of genes to ecosystems, available for researchers, stakeholders and policymakers alike.

## Methods

### Sampling and morphological identification

**Figure 2:**
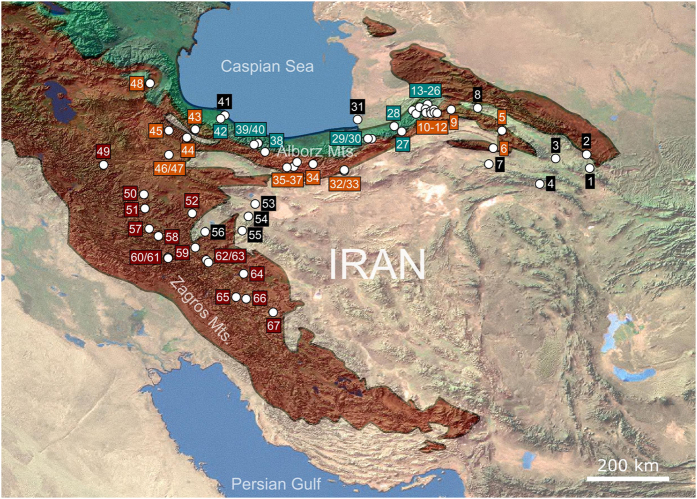
Overview of sampling localities. Shown are all localities where living amphipods have been found. Red area: Irano-Anatolian biodiversity hotspot; green area: Caucasus biodiversity hotspot. Localities are color coded in green (Alborz Mountains, in Caucasus biodiversity hotspot), orange (Alborz, Irano-Anatolian), red (Zagros, Irano-Anatolian) and black (Central Iranian Plateau or brackish water localities at the Caspian Sea; 31 and 41). The map was created using DIVA-GIS 7.5 (www.diva-gis.org).

We sampled 154 freshwater sites throughout the Alborz Mountains, Zagros Mountains and the Central Iranian Plateau, covering montane, arid and semi-arid areas. Most of the sampling sites are situated in the Irano-Anatolian and Caucasus biodiversity hotspots (Table 1 and Fig. 2). Amphipod specimens were found and collected from 67 of the 154 localities (43.5%) using dipping nets. Furthermore, two brackish water sites (Gomishan lagoon; GLA, and, Chaf lagoon; TCH) were visited to collect amphipod specimens as outgroups for the molecular analyses. Sampling was performed on macrophytes in the water, beneath stones and within sand. Freshly collected samples were immediately preserved in 96% ethanol and morphologically identified based on the most validated and recent freshwater amphipod keys^35^,^41^,^58^,^59^,^60^ as well as original species descriptions.

### DNA extraction, PCR, sequencing

Pereopods from one side of the body were used for DNA extraction by a salt precipitation method (modified after^61^, following^27^) and for at least two individuals per locality. The standard DNA barcoding locus—a 658 bp fragment of the Cytochrome c oxidase subunit I gene (*COI*)—was amplified using the modified primer pair LCO1490-JJ and HCO2198-JJ^62^. Polymerase chain reaction (PCR) was carried out in a total volume of 25 μL containing 2.5 μL 10X PCR buffer, 2.5 μL dNTPs (2 mM), 0.125 μL of each primer (100 pmol/μL), 0.125 μL of Hotmaster *Taq* (5 U/μL, 5 PRIME GmbH, Hamburg, Germany), 1 μL of template DNA (20–60 ng/μL) and molecular grade water. For samples which did not amplify under standard PCR settings, illustra PuReTaq Ready-To-Go PCR beads (GE Healthcare, Freiburg, Germany) were used with the following protocol: 0.125 μL of each primer, 1 μL of DNA, filled up to 25 μL with water. The PCR setting for *COI* amplification was: initial denaturation at 94 °C for 60 s; 36 cycles of denaturation at 94 °C for 30 s, annealing at 51 °C for 45 s, extension at 65 °C for 60 s; final extension at 65 °C for 5 min. We used a similar PCR setting for illustra PuReTaq Ready-To-Go PCR beads, with only the extension temperature adjusted to 72 °C. As an additional nuclear marker, the ribosomal DNA (rDNA) locus 28S was amplified. The 28S rDNA PCR reaction mix was the same as for *COI*, containing the primer pair 28Sa and 28Sb^63^. PCR settings were: initial denaturation at 94 °C for 60 s; 36 cycles of denaturation at 94 °C for 30 s, annealing at 48 °C for 45 s, extension at 65 °C for 60 s; final extension at 65 °C for 5 min. PCR setting in using illustra PuReTaq Ready-To-Go PCR beads was similar, but the extension temperature was adjusted to 72 °C. For DNA sequencing, 10 μL of PCR product was enzymatically purified with 0.5 μL ExoI (20 U/μL) and 1 μL FastAP (1 U/μL) (both Thermo Fisher Scientific, Schwerte, Germany). The reaction mix was incubated at 37 °C for 25 min and 85 °C for 15 min. The purified products were sequenced at GATC-Biotech (Cologne, Germany), Macrogen Inc. (Korea) or on an ABI 3130xl sequencer (Dept. of Receptor Biochemistry, Faculty of Chemistry, Ruhr University Bochum). Sequence chromatograms were edited and assembled in Geneious 6.0.6^64^. The *COI*-alignment was constructed using the MUSCLE algorithm plugin in Geneious with eight iterations^65^. The 28S rDNA alignment was generated using the Geneious MAFFT-plugin^66^, automatically selecting for the most appropriate algorithm.

### Molecular species delimitation

We used four molecular species delimitation approaches to delineate freshwater amphipod species with the standard barcode marker *COI*^67^. Our delimitation strategy consists of approaches based on three different delineation concepts: i) distance-based (ABGD), ii) network-based (SP), and iii) topology-based (bPTP, bGMYC). This combination was applied thus to compare results of conceptually different methods and to lower difficulties when relying on single parameter estimates only, i.e. genetic distance thresholds, topology-based transition points or connection limits in haplotype networks to distinguish intra- and interspecific diversity.

The Automatic Barcode Gap Discovery (ABGD^68^) method is based upon pairwise genetic distance calculations. Sequences are semi-automatically grouped in order that distances between sequences of two groups are always larger than a certain genetic distance threshold value, i.e. the barcode gap^68^. We tested the *COI* dataset with a combination of ABGD settings within the parameter range of Pmin = 0.001, Pmax = 0.08–0.10 and gap width = 0.5–0.8. A Kimura-2-parameter (K2P) corrected genetic distance matrix was used as it is the standard model proposed for DNA barcoding analyses^69^. K2P-distances were calculated using MEGA v6.0^70^.

The reversed Statistical Parsimony (SP) method^71^ delineates species based on the network topology. The calculation of a statistical parsimony network was performed in TCS v1.21^72^. Sequences were collapsed into haplotypes and connected based on a given connection probability of parsimony. If mutational steps between two haplotypes exceed the connection probability, i.e. a certain number of mutational steps, the haplotypes are clustered into two separate networks (or putative species). A 95% connection probability threshold was applied to delineate putative species.

The Bayesian Poisson Tree Process (bPTP)^73^ model approach delineates species based on a topology. The bPTP webserver (http://www.species.h-its.org) was used with a *COI* topology produced by BEAST v1.8.2^74^ as an input tree. The bPTP method was run under default settings. BEAST settings were computed in BEAUTi v1.8.2^74^: 30 million chain length, sampling each 3000th tree, standard coalescent model, GTR + G + I substitution model with four gamma categories and a strict clock. The GTR + G + I model was proposed by model selection in MEGA v6.0^70^ and jModeltest^75^. Appropriateness of parameters (effective sample size >200) was tested with Tracer v1.6^76^. Results were visualized with TreeAnnotator v1.8.2^77^ with a 10% burn-in rate, posterior probability of 0.9 and under the maximum clade credibility option for the consensus tree.

The Bayesian General Mixed Yule-Coalescent (bGMYC) model approach is conceptually similar to bPTP, but needs an ultrametric input tree^78^. The run parameters were mcmc = 100,000, burn-in: 90,000 and thinning = 100.

A Neighbor-joining (NJ) tree was calculated using MEGA based on K2P-distances and branch support assessed using 2000 bootstrap replicates. The 28S rDNA network was calculated in SplitsTree^79^ using uncorrected p-distances.

### Ethical approval

All applicable international, national, and/or institutional guidelines for the care and use of animals were followed. No experiments were done on living animals in this study. The Research and Ethics Committee of the College of Science, University of Tehran approved the experimental protocol.

## Additional Information

**Data Availability**: COI sequences: GenBank accession numbers: KT778323–KT778506. 28S rDNA sequences: GenBank accession numbers: KT827482–KT827554, KU513423–KU513436. Trees have been deposited in TreeBASE under submission number 18324. Voucher specimens and their comparative material are deposited at Zoological Museum, University of Tehran (ZUTC).

**How to cite this article**: Katouzian, A.-R. *et al.* Drastic underestimation of amphipod biodiversity in the endangered Irano-Anatolian and Caucasus biodiversity hotspots. *Sci. Rep.* **6**, 22507; doi: 10.1038/srep22507 (2016).

## Supplementary Material

Supplementary Information

## Figures and Tables

**Table 1:** List of sampling localities.

St. No.	St. Name	Abbreviation	GPS Coordinates		Altitude (m)	Date	N (COI)	N (28S rDNA)	Lineage
			N	E					
1	Baghe Keshmir	BK	35.76703	60.65006	1146	13/4/2013	4	1	Gk28
2	Mazdavand	MZD	36.15142	60.54992	1004	13/4/2013	2	1	Gk28
3	Meydan-e-Tir	MTR	36.03658	59.67403	1152	15/4/2013	4	0	Gk28
4	Forsheh Spring	FSH	35.32639	59.21533	1227	17/6/2013	2	1	Gk33
5	Darab Spring	DA	36.81758	58.14131	1592	12/4/2013	4	3	Gk18/Gk27
6	Gelabad	GLA	36.34	57.89306	1392	12/4/2013	2	2	*Gammarus lacustris* s. str.
7	Sheshtamad	SHT	35.89761	57.73717	1732	12/4/2013	2	2	Gk34
8	Baba Aman Spring	CBA	37.48483	57.43481	1059	17/6/2013	2	2	Gk19
9	Jozak Spring	JZ	37.42347	56.68867	1135	17/6/2013	2	2	Gk20
10	Mirzabayloo Old Qanat	QMB	37.35139	56.24314	1263	16/6/2013	2	2	Gk29
11	Shoor Cheshmeh	SHC	37.35794	56.20211	1377	16/6/2013	8	5	Gk21/Gk29
12	Yaghtikalan Spring	YQK	37.40081	56.19892	1603	15/6/2013	2	1	Gk13
13	Degarmanli Midstream	DGM	37.42472	56.14597	1273	15/6/2013	4	0	Gk29
14	Degarmanli Upstream	BDG	37.41764	56.13083	1347	15/6/2013	4	2	Gk29
15	Karkooli Spring	K	37.35175	56.08514	1731	16/6/2013	3	1	Gk3
16	Zoghali Spring	CHZ	37.43733	56.07675	1406	15/6/2013	1	0	Gk16
17	Yeki Barmagh Spring	YB	37.37456	56.04892	2027	16/6/2013	3	2	Gk3
18	Lohondor Water Reservoir	LH	37.56106	55.96781	1036	13/6/2013	3	1	Gk16
19	Chenar Spring	PTG	37.373	55.96986	743	16/6/2013	2	2	Gk3
20	Golestan Waterfall	AG	37.37317	55.96975	842	16/6/2013	3	1	Gk3
21	Janlar Stream	JLR	37.43783	55.95894	1610	14/6/2013	3	1	Gk3
22	Chatalghabagh Spring and Cave	QCQ	37.45711	55.93039	1398	16/6/2013	2	2	Gk3
23	Chatalghabagh Stream	MCQ	37.46178	55.92889	1374	14/6/2013	4	1	Gk3
24	Karim Ilan Spring	KIL	37.49275	55.79522	1328	13/6/2013	4	0	Gk16
25	Loveh Waterfall	L	37.35336	55.66278	632	17/6/2013	4	1	Gk15
26	Aghsoo River	AQS	37.41003	55.57319	274	15/5/2013	4	2	Gk5
27	Alang Sofla	JR	36.84319	55.24869	1920	17/5/2013	2	2	Gk4
28	Shirabad Waterfall	ASH	36.97165	55.03743	245	15/5/2013	8	3	Gk6/Gk7
29	Charbagh	CHB	36.6008	54.4362	2756	15/5/2013	3	0	Gl1
30	10 Km Charbagh	TCB	36.61878	54.32314	2149	15/5/2013	2	1	Gl1
31	Gomishan Lagoon	GMS	37.14914	54.00272	1	13/5/2013	2	2	*Gammarus aequicauda*
32	Ahovan Pass	GHV	35.70031	53.61769	1616	10/4/2013	2	1	Gk17
33	Ahovan	AHV	35.68033	53.61556	1557	10/4/2013	3	2	Gk17
34	Tange Vashi Stream	TVS	35.86928	52.7245	2275	21/5/2012	2	1	Gk30
35	Vana (Haraz River)	V	35.9264	52.27224	1435	16/9/2013	2	1	*Gammarus lacustris* s. str.
36	Lasem Waterfall	ALM	35.80625	52.18325	2620	17/4/2014	3	3	Gk31
37	Ala Spring	CHA	35.77694	51.98856	2536	17/4/2014	2	2	Gl2
38	Harijan	HJ	36.24044	51.33253	2451	11/6/2014	4	1	Gk14
39	Kelardasht	KLD	36.47353	51.14263	1364	15/5/2012	3	1	Gk1
40	Sardab River	SAR	36.43769	51.04617	2133	11/6/2014	0	0	
41	Chaf Lagoon	TCH	37.27503	50.20458	25	7/5/2012	2	1	*Obesogammarus acuminatus*
42	Divshel	DVS	37.17172	50.10822	196	12/6/2014	2	0	Gk17
43	Sardabkhani Spring	SRD	36.88376	49.37274	1511	28/6/2013	2	2	Gk35
44	Yash Bolagh	Y	36.62575	49.12861	1821	15/5/2014	2	1	Gk10
45	Taham Spring	THM	36.83547	48.60194	2232	16/5/2014	3	1	Gk9
46	Moosa Bolaghi Spring	MBQ	36.15875	48.6015	1877	16/5/2014	8	3	Gk8/*Gammarus balcanicus*
47	Moosa Bolaghi Stream	MBL	36.15814	48.59983	1867	16/5/2014	4	0	Gk8
48	Biledargh	BIL	38.17744	48.0577	1797	28/6/2014	6	3	Gk11/Gk12
49	Dare Dazoon	DDZ	35.86367	46.73032	2019	3/5/2014	2	2	*Gammarus lordeganensis*
50	Badr-o-Parishan	BPR	35.0271	47.8554	2180	24/5/2014	2	1	Gk32
51	Sarab-e-Fes	SAF	34.59835	47.91564	1565	5/5/2014	2	2	Gk23/Gk25
52	Bolagh Spring	BOQ	34.45694	49.25889	1912	2/8/2013	0	0	
53	Qom rood Nakhjiravan	QOM	34.72571	51.07429	846	19/8/2013	1	0	Gl4
54	Kahak	KHK	34.3928	50.86157	1447	15/5/2012	0	0	
55	Atashkooh Delijan	ATS	34.00262	50.69499	1578	4/6/2012	2	0	Gl4
56	Pahn Spring	CHP	33.92358	49.6562	2112	3/5/2014	1	0	Gk24
57	Sarab-e-PirHossein	SPH	34.04417	48.03444	1859	16/4/2013	2	2	Gk21
58	Sarab-e-Honam	SHO	33.80694	48.31333	1677	27/3/2013	2	1	*Gammarus hegmatanensis*
59	Qaladez Spring	QBZ	33.48758	49.35832	1917	4/5/2014	2	1	Gk24
60	Nozhian Waterfall	NZH	33.23105	48.57596	1429	2/5/2014	2	2	Gk21
61	Nozhan Waterfall Spring	MNZ	33.22333	48.57871	1400	2/5/2014	2	1	Gk21
62	Ab Sefid Waterfall	AAS	33.14586	49.68372	2024	3/5/2014	7	2	Gk32
63	Behesht River	BEH	33.06867	49.71861	1702	28/6/2014	2	1	Gl3
64	Soozanieh River	SZR	32.73656	50.75006	1964	3/7/2013	1	0	*Gammarus lordeganensis*
65	Rostam Abad Waterfall	RST	32.08867	50.51422	1770	1/6/2014	7	3	Gk22/*Gammarus lordeganensis*
66	Shalamzar Lake	DSH	32.02368	50.82342	2030	1/7/2013	0	0	
67	Morook	MRK	31.64411	51.58469	2378	21/8/2013	0	0	
							184	87	

From left to right station number, station name, station abbreviated name, coordinates, altitude, date of collection, number of individuals with *COI* data, number of individuals with 28S rDNA data and delimitation results respectively. Gk = *Gammarus komareki*, Gl = *G. lacustris*.
