# Supplementary material for: Drastic underestimation of amphipod biodiversity in the endangered Irano-Anatolian and Caucasus biodiversity hotspots
Source: Sci Rep. 2016 Mar 1;6:22507. doi: 10.1038/srep22507 (PMC4772388; doi:10.1038/srep22507)
Supplement: Supplementary Information [file srep22507-s1.pdf]

Drastic underestimation of amphipod biodiversity in the endangered Irano-Anatolian and Caucasus biodiversity hotspots

Ahmad-Reza Katouzian[1,*], Alireza Sari[1,*], Jan N. Macher[2], Martina Weiss[2], Alireza Saboori[3] Florian Leese[2], Alexander M. Weigand[2, 4]

Supplementary Information

Supplementary Figure 1- 28S rDNA network.
A: Overview of the 28S rDNA network showing the separation into the monophyletic *G. komareki* complex and the genetically more diverse lineages of the *G. lacustris* complex.
B: Details for the *G. komareki* complex. COI-groups and the respective specimen labels are indicated.
C: Details for the *G. lacustris* complex. COI-groups and the respective specimen labels are indicated.

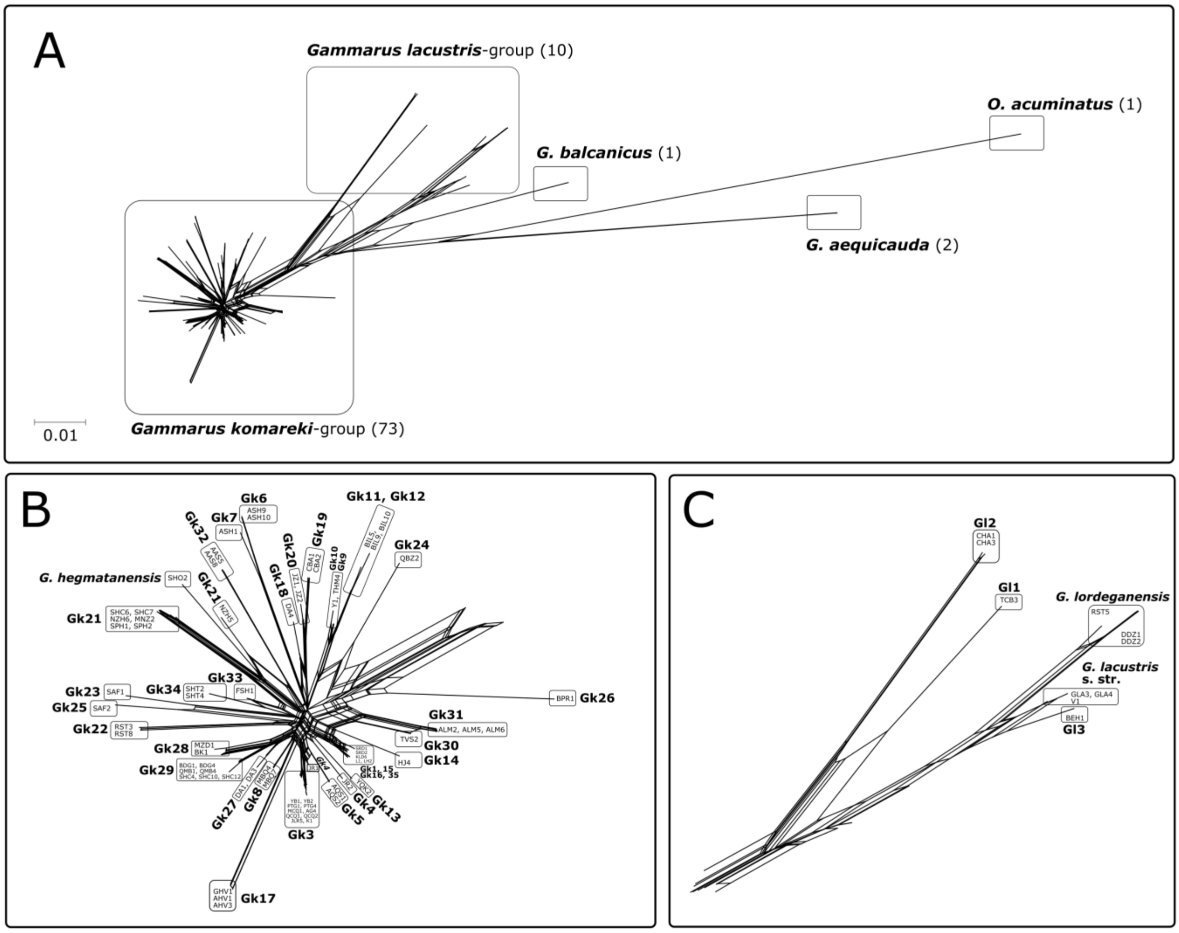

Supplementary Table 1- Type locality data for the morphologically identified species.

Species	Type Locality
Gammarus aequicauda	Donuslavscher lake, area of Evpatoria, 2 km from Donuzlav, Crimea
Gammarus balcanicus	Springs is Kolašin, prov. Crna Gora, Montenegro
Gammarus hegmatanensis	Gardaneh Asadabad spring, Hamadan province, Iran
Gammarus komareki	Village Bellovo near Pazaržik, Bulgaria
Gammarus lacustris	Vage, Selsvand, Norway
Gammarus lordeganensis	Barme-Lordegan spring, Lordegan, Chaharmahal Va Bakhtyari province, Iran
Obesogammarus acuminatus	Anzali wetlands, Gilan province, Iran
